# OA16 Lupus myocarditis causing acute heart failure-a rare complication of systemic lupus erythematosus - case report

**DOI:** 10.1093/rap/rkae117.016

**Published:** 2024-11-05

**Authors:** Rafia Waheed, Aqeel Anjum, Sharon Jones

**Affiliations:** University Hospital of Wales, Cardiff, United Kingdom; University Hospital of Wales, Cardiff, United Kingdom; University Hospital of Wales, Cardiff, United Kingdom

## Abstract

**Introduction:**

Systemic Lupus Erythematosus (SLE) is a multisystem autoimmune disorder that can affect any organ of the body. Cardiac involvement affecting any of three layers of cardiac tissue endocardium, myocardium, epicardium and pericardial serosa has been reported in greater than 50% of patients. Clinically significant myocarditis has been found in approximately 9 % of patients.

We present a case of 22-year-old female, known SLE, presenting with acute heart failure secondary to myopericarditis who was treated with intravenous methylprednisolone and belimumab. The aim is to highlight the clinical presentation, diagnostic approach and management of this challenging case.

**Case description:**

She presented to emergency department with acute onset shortness of breath, orthopnoea and intermittent pleuritic chest pain, with no signs of chest infection or risk factors for venous thromboembolism. She was known SLE (diagnosed in 2019, with arthralgia, positive ANA, anti-DsDNA, Raynaud’s, pericarditis, pleuritis, low C4, lupus nephritis-2021) and was on six monthly Rituximab, MMF 1 gm BD and hydroxychloroquine 400 mg OD. She had a history of congenital complete heart block secondary to maternal SLE antibodies exposure for which she had dual chamber pacemaker. Her latest echocardiogram in 2018 showed mild AR, MR with normal ejection fraction lupus nephritis (stage IV on renal biopsy 2021) causing CKD.

She had oxygen saturation of 95% on 3 litres oxygen, BP 160/100, raised JVP, bibasal crepitations on chest and normal neurological/abdominal examination.

Blood tests showed C3 0.91, C4 0.22, raised dsDNA 40.8, anti-ENA negative, anti-cardiac muscle antibodies, normal CK, Troponin 31 and BNP 56725. She also had AKI on CKD, creatinine 190 baseline 110, urine dip positive for blood and protein with urine PCR 170. ECG showed LBBB. CXR showed small right-sided pleural effusion with increased interstitial markings. She was treated for pulmonary oedema with intravenous diuretics and nitrates. TOE confirmed severe left ventricular systolic dysfunction (EF 20%), mechanical desynchrony, mildly thickened mitral valves, mild aortic regurgitation, dilated aortic root at 40 mm, chronically thickened RV pacing lead, and severely stenotic tricuspid valve.

After MDT discussion she was managed as myopericarditis secondary to SLE with pulse intravenous methylprednisolone followed by prednisolone 40 mg OD. Her SLEDAI was 6 and had BILAG 2004 cardiac A renal B manifestations. PET-CT scan didn’t show any endocarditis or aortitis. Patient preferred Belimumab over Cyclophosphamide (due to fertility concerns) hence was added to her treatment and steroids were tapered over 4 months. There was an excellent clinical and serological response.

**Discussion:**

Myocarditis is a rare but life-threatening condition, it has significant mortality and morbidity in around 50% of cases. The pathogenesis is not clear but it is thought to be immune mediated with complement activation. It is important to rule out other causes of myocarditis like viral, toxic, drug-induced and familial cardiomyopathy. Although cardiac MRI is considered a good non-invasive imaging modality for the diagnosis of myocarditis, our patient had a PET-CT given the presence of pacing leads and previous history of infective endocarditis. There are no standard guidelines for the management of lupus induced myocarditis, most cases are treated with pulsed steroids followed by cyclophosphamide, azathioprine, mycophenolate mofetil and rituximab. It is difficult to conduct clinical trials because of rarity of condition, so it is important to collect cases from across the world. The timely recognition and early steroid administration are imperative in SLE-related myopericarditis with cardiomyopathy to prevent the mortality associated with this condition.

Our patient developed end-stage renal failure a few months later, with a renal biopsy showing class 4 lupus nephritis and was started on regular haemodialysis. She was also started on cyclophosphamide with ovarian protection with a view that it might help recover her renal function. Previously she had refused cyclophosphamide in 2021 at her first presentation with Lupus nephritis. Unfortunately, she failed multiple attempts of egg retrieval.

Her cardiology function failed to improve on repeat echocardiograms and subsequently, she had external LV pacing lead placed using VATS due to occlusion of left subclavian and brachiocephalic veins. She had multiple admissions in one year for shortness of breath and haemodialysis access issues which affected her education. Moreover, despite her father being worked up as a live donor for her, she was unable to be activated on transplant register due to her poor ejection fraction.

**Key learning points:**

• This case highlights the importance of considering myopericarditis in SLE patients presenting with chest symptoms. It is important to have a high index of suspicion to prevent high mortality and morbidity associated with this condition. Early diagnosis and appropriate management are crucial to prevent long-term cardiac complications. However, it is important to bear in mind the side effects of aggressive management including myelosuppression, infections and effects on fertility.

• A multidisciplinary approach including cardiologists, rheumatologists and nephrologists is important for appropriate management. It is imperative to engage patients in all treatment decisions and it is even more relevant in the case of a young female with treatment resistant SLE, where preserving fertility is important and patients should be helped and supported in making these difficult decisions.

• SLE impacts physical, social and psychological aspects of a patient’s life. An integrated, patient centred management approach should be used to improve the quality of life of patients facing the challenges of this chronic relapsing and remitting illness.

